# Automated and Reproducible Detection of Vascular Endothelial Growth Factor (VEGF) in Renal Tissue Sections

**DOI:** 10.1155/2019/7232781

**Published:** 2019-03-19

**Authors:** Nayana Damiani Macedo, Aline Rodrigues Buzin, Isabela Bastos de Araujo, Breno Valentim Nogueira, Tadeu Uggere Andrade, Denise Coutinho Endringer, Dominik Lenz

**Affiliations:** ^1^University Vila Velha, Pharmaceutical Sciences, Vila Velha, Brazil; ^2^Department of Morphology, Federal University of Espírito Santo, Vitória, Brazil; ^3^Faculty of Medicine Carl Gustav Curav–Technische Universität Dresden (TUD), Dresden, Germany

## Abstract

**Background:**

Manual analysis of tissue sections, such as for pathological diagnosis, requires an analyst with substantial knowledge and experience. Reproducible image analysis of biological samples is steadily gaining scientific importance. The aim of the present study was to employ image analysis followed by machine learning to identify vascular endothelial growth factor (VEGF) in kidney tissue that had been subjected to hypoxia.

**Methods:**

Light microscopy images of renal tissue sections stained for VEGF were analyzed. Subsequently, machine learning classified the cells as VEGF^+^ and VEGF^−^ cells.

**Results:**

VEGF was detected and cells were counted with high sensitivity and specificity.

**Conclusion:**

With great clinical, diagnostic, and research potential, automatic image analysis offers a new quantitative capability, thereby adding numerical information to a mostly qualitative diagnostic approach.

## 1. Introduction

The manual analysis of tissue sections, such as the analysis performed for pathological diagnosis, requires an analyst with substantial knowledge and experience [1, 2]. Usually, the tissue sections are stained to unequivocally identify nuclei and cytoplasm [3]. In most biological tissue analyses, e.g., immunohistochemistry, cells are counted manually [4].

However, manual tissue analysis and cell counting are considered subjective, tedious, and time consuming, resulting in intra-analyst variance [4–8]. In pathology, a rather qualitative diagnostic science, the need for quantitative analysis of histopathological images has been recognized [9], and pathologists have been aiming to combine the quantitative nature of the analysis with reproducibility and precision [10].

For biological analyses of tissue, many cells should be observed to correlate a certain cellular morphology with a biological process. In terms of image analysis of biological samples, many images are needed [11]. The importance of reproducible image analysis of biological samples, i.e., an automated process for identifying objects of interest and performing a subsequent quantitative per-object analysis, is steadily being recognized by the scientific community [11, 12].

The use of software for automated analysis of tissue enables fast analysis and cell counting [4]. The available software includes CellProfiler (CP) and CellProfiler Analyst (CPA) for image analysis and statistical processing, respectively. Both programs are freely available. CP allows automated cellular identification and the analysis of hundreds of parameters to gain a plethora of information about intensity, morphology, and texture [13]. Furthermore, the software offers simultaneous analysis of different images (Carpenter et al., 2006) and a reproducible analysis [14, 15]. The CPA software has a machine learning-based classifier that can be used, e.g., to identify and count different cell types or cells in different phases of the cell cycle [13].

The classification and subsequent counting of cells using machine learning are steadily gaining scientific attention [16]. As a further advantage, the use of open software allows the verification of results by almost every laboratory in the world [17].

The aim of the present study was to employ image analysis and subsequent machine learning to identify vascular endothelial growth factor (VEGF) in kidney tissue that had been subjected to hypoxia.

## 2. Methods

### 2.1. Ethical Approvement and Consent

Ethical approval was obtained by the Federal University of Espírito Santo (UFES) (CEUA/UFES (Protocol no. 050/2013)).

### 2.2. Preparation of Slides

#### 2.2.1. Immunohistochemistry Staining

Four-micrometer serial paraffin sections of the kidney were stained with monoclonal mouse anti-rat VEGF (ab1316) antibody (Abcam, UK, 1 : 200). The staining was visualized with the peroxidase reaction with 3,3′-diaminobenzidine tetrahydrochloride (DAB; Sigma Chemical Co., USA). The specimens were then lightly counterstained with Mayer's hematoxylin, dehydrated, and mounted in xylene under glass cover slips. The human placenta was used for the positive control sample, while the sample material incubated with antibody diluent only was used for the negative control.

#### 2.2.2. Material

Seven slides with tissue sections were used for the present study. The tissue was hypoxic kidney tissue sections of Wistar rats that had been subjected to hypoxia.

#### 2.2.3. Animals

The rats were randomly divided for the experimental set-up (control or sham and hypoxic kidneys). During the entire experiment, the cages were housed in a controlled environment: temperature (20-22°C), light/dark cycle (12 h), and ventilation at UFES animal facility. The animals had free access (*ad libitum*) to water and food (Labina, Purina®).

In order to induce hypoxia, intraperitoneal (ip) administration of ketamine and xylazine (1.0 ml/kg) was given according to the weight of each animal. Once the pain reflexes were absent (tested by squeezing the toes with tweezers), the rat was placed on a temperature-controlled heating surgical table (37°C) and had its arms and legs fixed by tapes. Immediately before the operation, Temgesic® (sc) was administrated (0.1 mg/kg). Following disinfection and shaving of the skin, an incision of approximately 2.0 cm was made in the abdomen. The visceral organs were placed by side and covered with surgical gaze moisture in NaCl 0.9%. The kidney was carefully exposed and decapsulated, and the entire renal pedicle (artery, vein, and nerve) was gently isolated from the adjacent tissues close to its take-off from the abdominal aorta with fine 45° angled forceps (tip width 0.40 mm, 9 cm) and fine curved serrated forceps (tip width 0.60 mm, 7 cm). Thoroughly, the pedicle was faintly suspended assisted by a blunt hook 12 cm and a nonabsorbable sterilized 4/0 silk black suture was placed slowly under it by using 45° angled forceps as a leading guide. The blood flow occlusion was done by ligating the pedicle for 40 minutes, causing the ischemia phenomena. Successful obstruction is confirmed by a color change from vivid red to pale, at first instance, and later dark red. During this time, the incision was temporarily closed to prevent drastic temperature changes and dehydration. Additionally, 100-200 *μ*l of prewarmed (37°C) NaCl was given.

The rats were sacrificed at the 3rd day after they underwent surgeries (control or hypoxia) with overdose of ketamine (10.0 mg/ml) and xylazine (2.0 g/ml) solution.

### 2.3. Imaging

Images of the slides were taken using a ZeissAxioVert. A1 microscope (40x objective) equipped with a digital camera (AxioCam MRC Zeiss). Images were manually taken without defining an exposure time; no filters were used. The NA of the objective was 0.85. Images were saved in ∗.tiff-format using appropriate names.

### 2.4. Image Analysis

Image analysis was conducted using CP (version 2.1.1) [18].

CellProfiler has different modules, and the combination of different modules used to conduct image analysis is called a pipeline.  Table 1 depicts the pipeline used for the present study, which contains eight modules.

**Figure 1:**
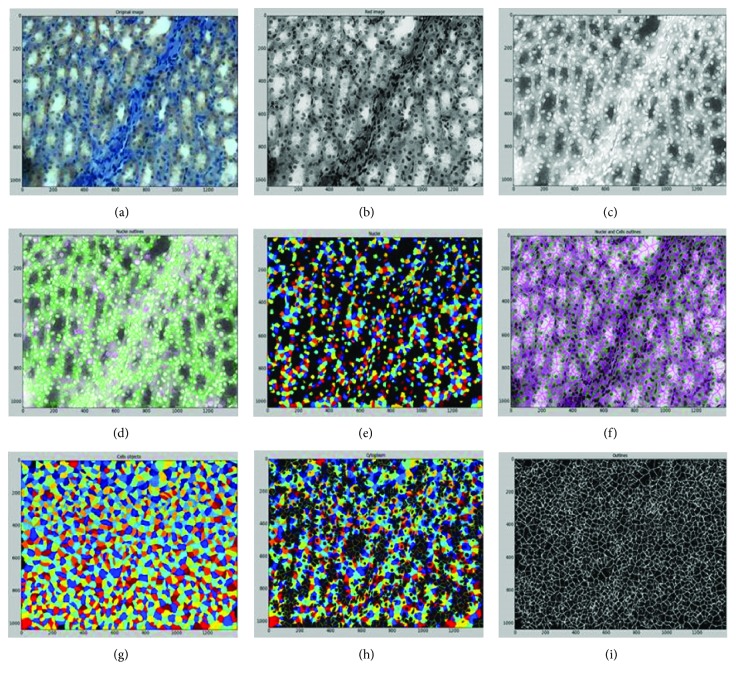
Identification of the objects: the pipeline of CellProfiler. (a) Original image. (b) Image (a) converted to grayscale. (c) Image (b) with inverted intensities. (d) Identified nuclei. (e) Identified nuclei. Different colors indicate different objects. (f) Identified cells. Different colors indicate different objects. (g) Identified cytoplasm (i.e., cellular area minus nuclear area). Different colors indicate different objects.

VEGF protein is expressed in the cytoplasm; therefore, VEGF is the only object that is the subject of analysis.  Figure 1 shows the identification or creation of the three objects (nucleus, cell, and cytoplasm).

### 2.5. Machine Learning

After the image analysis was finished, the data were exported to a database (SQLite format) for further analysis using CPA (version 2.0), which was previously downloaded from the homepage of the developers [19].

The machine learning process was supervised, i.e., the user assembled the training set actively. To this end, single objects displayed by the CPA software showed single identified objects. By double-clicking a single object, the entire image was displayed with the object of interest being highlighted. This process enabled a control of every identified/classified object.

**Figure 2:**
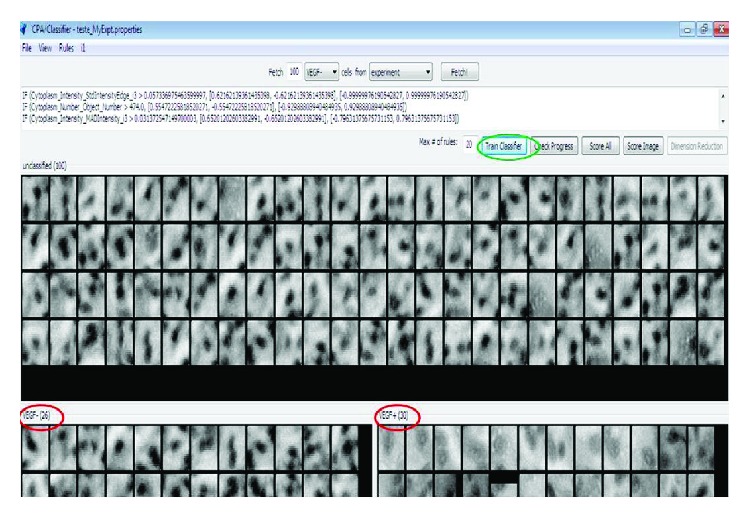
Interface of CellProfiler Analyst. Objects on the left side of the training set were classified as VEGF^−^, and objects on the right side were considered VEGF^+^.

**Figure 3:**
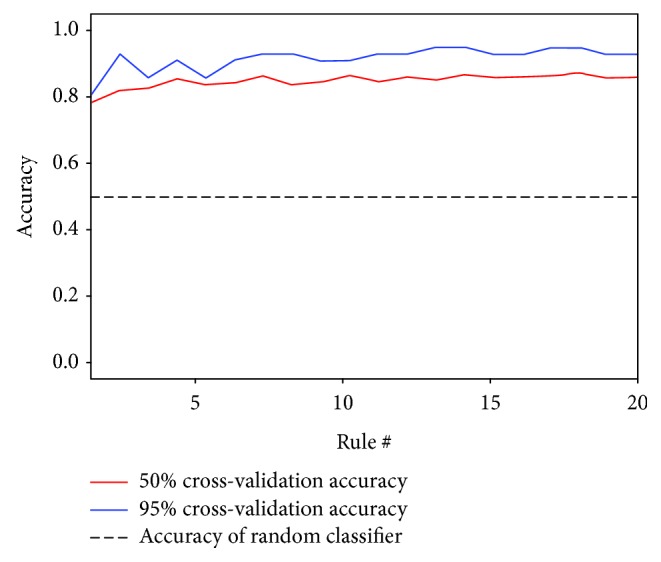
Progress of machine learning.

The classification was based on grouping the objects based on their similarities, i.e., VEGF^+^ cells were grouped, and VEGF^−^ cells were grouped. Initially, randomly shown objects were separated (VEGF^+^ and VEGF^−^ cells) to create a training set (Figure 2). With the objects distributed into their respective classes, the “train classifier” tool was activated to initiate the machine learning process (boosting) with the goal of automated identification and subsequent counting of the objects of the different classes (Sommer and Gehrlich, 2013). After adding new cells to the training set, the “train classifier” tool was used to improve the automated classification. Another tool to evaluate the progress of the machine learning is the “check progress” tool (Figure 3). An accuracy above 80% is considered appropriate [20].

**Figure 4:**
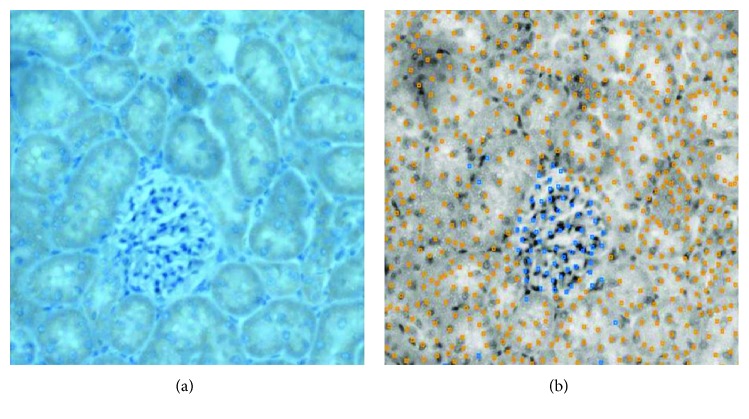
“Score image” classifier of CellProfiler Analyst. (a) Original image stained with Harris hematoxylin and DAB. Orange indicates VEGF^+^ cells. (b) Classification of the “score image” tool. Objects with blue points are classified as VEGF^+^, and objects with orange points are identified as VEGF^−^.

The machine learning process can also be assessed with the “score image” tool, which shows the classification of the machine of each identified object on an entire image (Figure 4).

## 3. Results

More than 18,000 objects were identified; approximately 74% were classified as VEGF^+^ and 26% as VEGF^−^. The sensitivity and specificity are listed in Table 2. The positive predictive value (PPV) was 0.95 and the negative predictive value (NPV) was 0.88.

**Figure 5:**
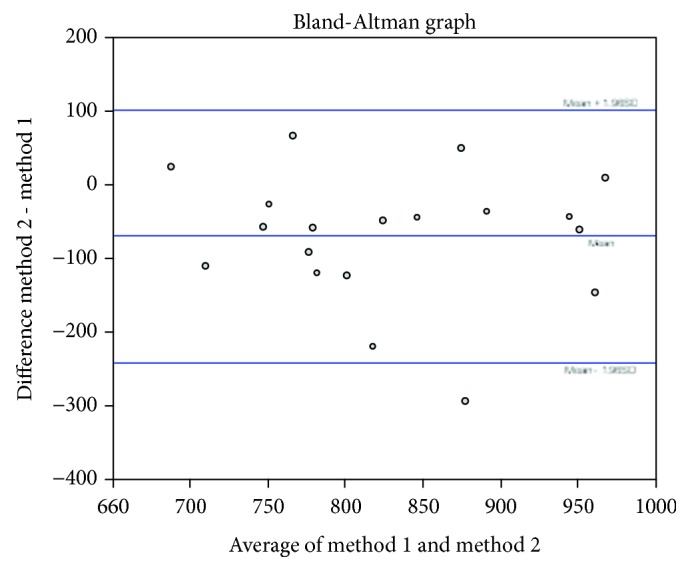
The Bland-Altman test to compare the manual and automated counts.

A Bland-Altman test (Figure 5) was used to assess the similarity between the manual and automated counts. The mean difference between the two counts was -70 (middle line). A large number of events were within ±1.96 standard deviations of the average (lower and upper lines). There were no systematic biases in the comparisons because there were both positive and negative results, i.e., the automated count was either the same, higher, or lower than the manual count.

**Figure 6:**
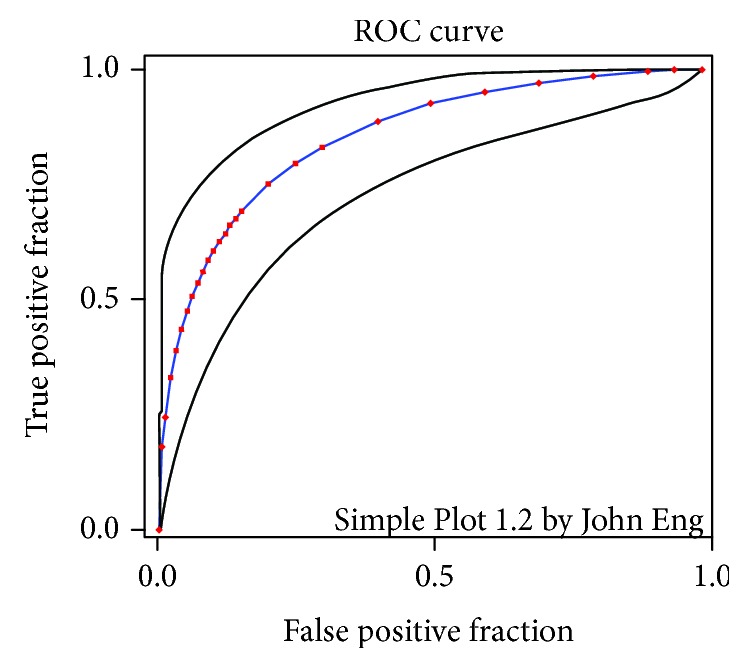
ROC curve to evaluate the machine learning process.

The receiver operating characteristic (ROC) curve (Figure 6) was generated to assess the machine learning process. Initially, 10 objects from each group were added to the training set. Ten more objects were added to each class of the training set, and the sensitivity was recalculated. This process was repeated until the training set contained 100 objects in each group. The area under the curve (AUC) was 86%.

## 4. Discussion

Automated analysis of cells and/or tissue is gaining scientific importance. Görtler et al. [21] stated about the function of automated analysis as a tool to enhance medical doctors' work. Kayser et al. [22] highlighted the importance of automated analysis in time-related measurements in order to describe and interpret biological functions in living organisms at the cellular level. An increasing number of studies have highlighted the importance of automated image analysis and subsequent image classification [23–26]. According to Deroulers et al. [27], quantitative histology is a promising new area that combines cellular morphometry, computers, and statistical analysis of tissues. A quantitative approach is important not only for clinical and diagnostic applications (e.g., to reduce intra-analytic variations) but also for understanding specific diagnoses and for research purposes [9].

Automated quantitative image analysis has recently gained substantial attention [28]. This new approach notably differs from most of the microscopy approaches used in the last few years [29]. This computational approach is effective and able to objectively analyze images and subsequently recognize patterns. According to Shamir et al. (2008), the machine learning-based recognition of patterns allows the differentiation of different groups of cells.

Krajewska et al. [30] characterized cellular processes associated with cell death using image analysis. Dordea et al. [17] automatically quantified rat retinal ganglion cells using the free open-source software programs CP and CPA. The authors found that the automated method made their analyses approximately 10 times faster.

For the present study, the programs CP and CPA were used because the software offers image analysis, machine learning, and subsequent classification (i.e., diagnosis) without the need to download and install further plug-ins and is relatively easy to use (Carpenter et al., 2006).

The present study demonstrated that automatic image analysis can be used to identify and quantify VEGF in tissue. Other studies identified HIF1a-positive cells [31] and TUNEL-positive cells [32] in renal tissue sections. Diem et al. [4] used automatic image analysis to count CD4^+^ and CD8^+^ T cells in human tissue and stated that even for images with a high cell density the automated counting was approximately 10 minutes faster than manual counting. Notably, automatic counting provides faster processing and analysis of samples. Images appropriately saved on hard disks can be reanalyzed numerous times, which may be important for forensic purposes.

## 5. Conclusion

With great clinical, diagnostic, and research potential, automatic image analysis offers a new quantitative capability, thereby adding numerical information to a mostly qualitative diagnostic approach.

This technique, as already described, provides the user with a fast, accurate, and reproducible analysis and is capable of greatly reducing intra-analytic variability.

## Figures and Tables

**Table 1:** Pipeline programmed for image analysis with CP.

Module	Operation
(1) LoadImages	Identify and load images in .tiff
(2) ColorToGray	Conversion method: split
(3) Morph	Operation: invert
(4) IdentifyPrimaryObjects	(a) Identify an object of interest: core (b) Maximum and minimum area: 13-40 (c) Threshold strategy: adaptive (d) Threshold method: MCT
(5) IdentifySecondaryObjects	(a) Object name: cell (b) Method to identify the secondary objects: propagation (c) Threshold strategy: adaptive (d) Threshold method: kapur
(6) IdentifyTertiaryObjects	Object name: cytoplasm
(7) MeasureObjectSizeShape	Measurement object: cytoplasm
(8) MeasureObjectIntensity	Measurement object: cytoplasm

(1) Load user-defined images. (2) Convert the original images to grayscale images. (3) Invert intensities to have bright nuclei. (4) Identify the primary object of interest (in this case, the nucleus). (5) Identify the secondary object (in this case, the entire cell). (6) Create the tertiary object (cytoplasm) by subtracting the primary object from the secondary object, i.e., subtracting the nucleus from the cell. (7) Analyze morphologic parameters in the object called cytoplasm. (8) Calculate intensity parameters in the object called cytoplasm.

**Table 2:** Results of the machine learning-based classification in terms of sensitivity and specificity.

Sample ID	Sensitivity	Specificity	% VEGF^+^	% VEGF^−^
1	0.86	0.92	69%	31%
2	0.95	0.88	79%	21%
3	0.98	0.89	75%	25%
4	1	0.84	83%	17%
5	0.97	0.87	59%	41%
6	0.99	0.91	83%	17%
7	1	0.81	73%	27%

## Data Availability

The data used to support the findings of this study are available from the corresponding author upon request.

## Abbreviations

AUC: Area under the curve
CP: CellProfiler
CPA: CellProfiler Analyst
ROC: Receiver operating characteristic
TUNEL: TdT-mediated dUTP-biotin nick end labeling
VEGF: Vascular endothelial growth factor.

## References

[1] Chan L. L., Kury A., Wilkinson A., Berkes C., Pirani A. (2012). Novel image cytometric method for detection of physiological and metabolic changes in *Saccharomyces cerevisiae*. *Journal of Industrial Microbiology & Biotechnology*.

[2] He L., Long L. R., Antani S., Thoma G. R. (2012). Histology image analysis for carcinoma detection and grading. *Computer Methods and Programs in Biomedicine*.

[3] Bigley A. L., Klein S. K., Davies B., Williams L., Rudmann D. G. (2016). Using automated image analysis algorithms to distinguish normal, aberrant, and degenerate mitotic figures induced by Eg5 inhibition. *ToxicolPathol.*.

[4] Diem K., Magaret A., Klock A., Jin L., Zhu J., Corey L. (2015). Image analysis for accurately counting CD4+ and CD8+ T cells in human tissue. *Journal of Virological Methods*.

[5] Nelissen B. G. L., van Herwaarden J. A., Moll F. L., van Diest P. J., Pasterkamp G. (2014). SlideToolkit: an assistive toolset for the histological quantification of whole slide images. *PLoS One*.

[6] Prichard J. W., Davison J. M., Campbell B. B. (2015). TissueCypher™: a systems biology approach to anatomic pathology. *Journal of Pathology Informatics*.

[7] Wang F., Kong J., Cooper L. (2011). A data model and database for high-resolution pathology analytical image informatics. *Journal of Pathology Informatics*.

[8] Zerbe N., Hufnagl P., Schlüns K. (2011). Distributed computing in image analysis using open source frameworks and application to image sharpness assessment of histological whole slide images. *Diagnostic Pathology*.

[9] Gurcan M. N., Boucheron L. E., Can A., Madabhushi A., Rajpoot N. M., Yener B. (2009). Histopathological image analysis: a review. *IEEE Reviews in Biomedical Engineering*.

[10] Eliceiri K. W., Berthold M. R., Goldberg I. G. (2012). Biological imaging software tools. *Nature Methods*.

[11] Kerz M., Folarin A., Meleckyte R., Watt F. M., Dobson R. J., Danovi D. (2016). A novel automated high-content analysis workflow capturing cell population dynamics from induced pluripotent stem cell live imaging data. *Journal of Biomolecular Screening*.

[12] Misselwitz B., Strittmatter G., Periaswamy B. (2010). Enhanced CellClassifier: a multi-class classification tool for microscopy images. *BMC Bioinformatics*.

[13] Hennig H., Rees P., Blasi T. (2017). An open-source solution for advanced imaging flow cytometry data analysis using machine learning. *Methods*.

[14] Buzin A. R., Pinto F. E., Nieschke K. (2015). Replacement of specific markers for apoptosis and necrosis by nuclear morphology for affordable cytometry. *Journal of Immunological Methods*.

[15] Tozetti P. B., Lima E. M., Nascimento A. M. (2014). Morphometry to identify subtypes of leukocytes. *Hematology/Oncology and Stem Cell Therapy*.

[16] Uhlmann V., Singh S., Carpenter A. E. (2016). CP-CHARM: segmentation-free image classification made accessible. *BMC Bioinformatics*.

[17] Dordea A. C., Bray M. A., Allen K. (2016). An open-source computational tool to automatically quantify immunolabeled retinal ganglion cells. *Experimental Eye Research*.

[18] Lamprecht M. R., Sabatini D. M., Carpenter A. E. (2007). CellProfiler: free, versatile software for automated biological image analysis. *BioTechniques*.

[19] Jones T. R., Kang I. H., Wheeler D. B. (2008). CellProfiler Analyst: data exploration and analysis software for complex image-based screens. *BMC Bioinformatics*.

[20] Jones T. R., Carpenter A. E., Lamprecht M. R. (2009). Scoring diverse cellular morphologies in image-based screens with iterative feedback and machine learning. *PNAS*.

[21] Görtler J., Kayser K., Borkenfeld S., Carvalho R., Kayser G. (2017). Cognitive algorithms and digitized tissue - based diagnosis. *Diagnostic Pathology*.

[22] Kayser K., Borkenfeld S., Carvalho R., Diejenouni A., Kayser G. (2016). How to analyze structure and function in tissue – based diagnosis?. *Diagnostic Pathology*.

[23] Chang H., Han J., Borowsky A. (2013). Invariant delineation of nuclear architecture in glioblastoma multiforme for clinical and molecular association. *IEEE Transactions on Medical Imaging*.

[24] Demir C., Gultekin S. H., Yener B. (2005). Augmented cell-graphs for automated cancer diagnosis. *Bioinformatics*.

[25] Kayser K., Radziszowski D., Bzdyl P., Sommer R., Kayser G. (2006). Towards an automated virtual slide screening: theoretical considerations and practical experiences of automated tissue-based virtual diagnosis to be implemented in the Internet. *Diagnostic Pathology*.

[26] West N. P., Dattani M., McShane P. (2010). The proportion of tumour cells is an independent predictor for survival in colorectal cancer patients. *British Journal of Cancer*.

[27] Deroulers C., Ameisen D., Badoual M., Gerin C., Granier A., Lartaud M. (2013). Analyzing huge pathology images with open source software. *Diagnostic Pathology*.

[28] Shamir L., Delaney J. D., Orlov N., Eckley D. M., Goldberg I. G. (2010). Pattern recognition software and techniques for biological image analysis. *PLoS Computational Biology*.

[29] Peng H. (2008). Bioimage informatics: a new area of engineering biology. *Bioinformatics*.

[30] Krajewska M., Smith L. H., Rong J. (2009). Image analysis algorithms for immunohistochemical assessment of cell death events and fibrosis in tissue sections. *Journal of Histochemistry & Cytochemistry*.

[31] Buzin A. R., Macedo N. D., de Araujo I. B. B. A. (2017). Automatic detection of hypoxia in renal tissue stained with HIF-1alpha. *Journal of Immunological Methods*.

[32] Macedo N. D., Buzin A. R., de Araujo I. B. B. A. (2017). Objective detection of apoptosis in rat renal tissue sections using light microscopy and free image analysis software with subsequent machine learning: detection of apoptosis in renal tissue. *Tissue and Cell*.

